# School hygiene and deworming are key protective factors for reduced transmission of soil-transmitted helminths among schoolchildren in Honduras

**DOI:** 10.1186/1756-3305-7-354

**Published:** 2014-08-04

**Authors:** José Antonio Gabrie, María Mercedes Rueda, Maritza Canales, Theresa W Gyorkos, Ana Lourdes Sanchez

**Affiliations:** Department of Health Sciences, Brock University, St. Catharines, Ontario Canada; School of Microbiology, National Autonomous University of Honduras (UNAH), Tegucigalpa, Honduras; Microbiology Research Institute, UNAH, Tegucigalpa, Honduras; Division of Clinical Epidemiology, Research Institute of the McGill University Health Centre, Montreal, Quebec Canada

**Keywords:** Soil-transmitted helminths, Geohelminths, Schoolchildren, Risk factors, Deworming, Hygiene, Cross-sectional study, Honduras

## Abstract

**Background:**

Among many neglected tropical diseases endemic in Honduras, soil-transmitted helminth (STH) infections are of particular importance. However, knowledge gaps remain in terms of risk factors involved in infection transmission. The aim of this study was to investigate risk factors associated with STH infections in schoolchildren living in rural Honduras.

**Methods:**

A cross-sectional study was conducted among Honduran rural schoolchildren in 2011. Demographic, socio-economic, and epidemiological data were obtained through a standardized questionnaire and STH infections were determined by the Kato-Katz method. Logistic regression models accounting for school clustering were used to assess putative risk factors for infection.

**Results:**

A total of 320 children completed the study. Prevalences for any STH and for *Ascaris lumbricoides*, *Trichuris trichiura* and hookworms were: 72.5%, 30.3%, 66.9% and 15.9%, respectively. A number of risk factors were identified at the individual, household, and school level. Boys were at increased odds of infection with hookworms (OR 2.33, 95% CI = 1.23-4.42). Higher socio-economic status in the family had a protective effect against infections by *A. lumbricoides* (OR 0.80, 95% CI = 0.65-0.99) and *T. trichiura* (OR 0.77, 95% CI = 0.63-0.94).

Low school hygiene conditions significantly increased the odds for ascariasis (OR 14.85, 95% CI = 7.29-30.24), trichuriasis (OR 7.32, 95% CI = 3.71-14.45), mixed infections (OR 9.02, 95% CI = 4.66-17.46), and ascariasis intensity of infection (OR 3.32, 95% CI = 1.05 -10.52).

Children attending schools not providing deworming treatment or that had provided it only once a year were at increased odds of ascariasis (OR 10.40, 95% CI = 4.39-24.65), hookworm (OR 2.92, 95% CI = 1.09-7.85) and mixed infections (OR 10.57, 95% CI = 4.53-24.66).

**Conclusions:**

Poverty-reduction strategies will ultimately lead to sustainable control of STH infections in Honduras, but as shorter-term measures, uninterrupted bi-annual deworming treatment paired with improvements in school sanitary conditions may result in significant reductions of STH prevalence among Honduran schoolchildren.

## Background

Five intestinal nematodes: *Ascaris lumbricoides*, *Trichuris trichiura*, *Strongyloides stercoralis* and the hookworms, *Ancylostoma duodenale* and *Necator americanus*, are collectively known as geohelminths or soil-transmitted helminths (STH) due to their ability to survive in the environment and be transmitted through faecally-contaminated soil [1, 2]. These geohelminthiases are among the 17 neglected tropical diseases of greatest public health concern [3, 4] because their health burden is particularly important in children and women of childbearing age [5, 6]. Of the five species, *S. stercoralis* is the most overlooked due to the low diagnostic sensitivity of the methods commonly used to detect STH [7, 8], but disseminated infections can be fatal, particularly in immunosuppressed individuals [9].

Transmission of STH is complex as it is determined by the dynamic interaction of a multitude of factors. Social structural determinants (*e.g*., poverty, lack of safe water, inadequate sanitation, and inaccessible health care) in combination with favourable ecological conditions (*e.g*., climate, temperature, soil, altitude, and precipitation) create the overall necessary landscape in which STH infections will thrive [10]. Once these helminthiases become endemic in human settlements, a low level of education–especially health education–, lack of awareness about their importance and transmission mechanisms, as well as cultural and behavioral practices among the population, contribute to their propagation. Understanding which transmission factors are operating in a particular endemic community is key for the effective control of these parasitoses [11], but given the complex web of socio-biological determinants at play [12, 13], identifying such risk factors might be challenging [14].

Although the exact number of people infected globally has yet to be determined, Pullan *et al*. recently estimated that a total of 1.45 billion people were infected with at least one STH species in 2010 [15]. In terms of school-age children, the WHO estimated that worldwide, in 2010, 173 million children requiring anthelmintic therapy were treated, for a global coverage of 28.2% [16]. This means that at that time there were more than 613 million school-age children living at risk of infection on the planet.

In Latin America and the Caribbean (LAC), there are 30 STH-endemic countries and, according to the Pan-American Health Organization (PAHO), in 2012, there were in the region 49.3 million pre-school and school-age children at risk of infection [17]. Currently, 2.6 million of these children (including 1,840,094 school-age children) are living in Honduras (http://apps.who.int/neglected_diseases/ntddata/sth/sth.html, accessed July 17, 2014), a lower-middle income Central American country in which 60% of the population lives in poverty (http://data.worldbank.org/country/honduras, accessed July 17, 2014). Undernutrition is prevalent among 32% and 13.7% of Honduran children living in rural and urban settings, respectively [18]. Furthermore, recent studies have shown that STH infections have a detrimental effect on the nutritional status of Honduran schoolchildren living in impoverished rural communities [19].

With the exception of large urban centres, due to generalized deficient sanitization and hygiene, lack of clean water, and inadequate access to health care, STH transmission in Honduras occurs throughout the year and across the country. Since 2001, the Honduran government has systematically tackled the challenge of decreasing the burden of these infections [10, 20]. Preventive chemotherapy through national deworming campaigns targeting school-age children (and more recently pre-school children) implemented by the Ministries of Health and Education with the technical support of PAHO/WHO has been the chief intervention, as is the case in all endemic countries [4]. Due to these efforts, it is likely that STH transmission has decreased throughout Honduras, but still a national prevalence between 20 and 50% is consistently reported [15]. In addition, recent studies have identified high-risk areas where STH endemicity is greater than 50% [20]. These data have underscored the need for epidemiological studies that help understand the transmission dynamics of these geohelminthiases in the country. The aim of the present study was to identify risk factors associated with STH transmission and infection intensity among primary schoolchildren residing in rural Honduras.

## Methods

### Ethical considerations

The present epidemiological study was nested within a parent study entitled “Gender and parasitic diseases: Integrating gender analysis in epidemiological research on parasitic diseases to optimize the impact of prevention and control measures” (Principal investigator, T. W. Gyorkos, McGill University, Canada). Both studies received institutional ethics approval by the three universities involved: Ethics Board of McGill University Health Centre, Montreal, QC (MUHC 10-175-PED, 23 November 2010), Ethics Board of Brock University, St. Catharines, ON (BU 10–161, 13 January 2011), and the Ethics Officer of the Master’s degree program in Zoonotic and Infectious Diseases, School of Microbiology of the National Autonomous University of Honduras (OF-MEIZ-001-2011, 10 February 2011). Approval was also obtained from community leaders, school authorities, and teachers, as well as from parents and legal guardians of eligible schoolchildren. Details of the consent and assent processes can be found in a related publication based on the same study population but looking at nutritional status and STH infection [19]. After obtaining parental consent, children with STH infections were treated by school teachers with a single dose of albendazole (chewable tablets of 400 mg) provided by the Ministry of Health.

### Study design, geographic area, and study population

Both the parent and the present epidemiological study were school-based, cross-sectional studies. Details on sample size calculations and field implementation have been described in a related report [19]. Briefly, the field work took place from January to March 2011, during which period nine primary schools located in rural communities within the Municipality of Catacamas, Department of Olancho, were visited: Colonia de Poncaya; Las Lomas de Poncaya; Las Parcelas; Corosito de Poncaya; Los Lirios; El Cerro del Vigía, El Hormiguero; Santa Clara; and Campamento Viejo. Schools, which had not administered deworming treatment in the last three months, were eligible for enrolment in the study. Schoolchildren specifically attending grades 3rd to 5th (usually aged 8–11 years) were invited to participate because infections tend to peak at these ages [2, 5, 10, 19]; they are also old enough to respond to survey questions and provide basic information. A total of 445 children constituted the eligible study population.

### Stool samples collection and parasite determination

One stool sample was required from each participant to determine presence and intensity of infection using the Kato-Katz method [21, 22]. With the help of their parents, children collected stool samples first thing in the morning the day of the interview and brought them to the research team within one hour of production. Samples were immediately placed in portable coolers to minimize parasite egg/larvae deterioration, transported to the National University of Agriculture’s laboratory in Catacamas, where they were refrigerated until smear preparation and examination for parasites the same day. The time from sample production to Kato-Katz smear preparations was approximately six hours and from the latter to microscopic examination was 30–60 minutes. Helminth eggs were identified by their characteristic features and systematically counted. Fecal egg counts were calculated for each parasite species by multiplying the numbers of eggs by a factor of 24, thus obtaining the number of eggs per gramme of stool (epg). Infection intensities were classified as light, moderate, or heavy based on the epg calculations, according to the WHO criteria [2]. The Kato-Katz method is not ideal for *Strongyloides stercoralis* diagnosis, but due to the field working conditions and time constraints, no attempts were made to implement more sensitive techniques such as Koga agar plate and Baermann, as carried out by other authors [23].

### Children’s demographic and epidemiological information

Demographic, socio-economic, and epidemiological data were collected by trained interviewers through individual, private, face-to-face interviews using a pre-tested standardized questionnaire in Spanish. Depending on the children’s ability to understand and answer questions, interviews lasted between 25 and 30 minutes. Data collected included: basic demographic information, household characteristics, hygiene practices, children’s chores and play activities; use of shoes or sandals; history of STH infections and deworming, as well as the level of awareness about intestinal parasites. Assessing awareness was preferred to simply measuring if children gave correct answers to independent questions, and as explained later, its assessment included if children thought they were at risk of acquiring parasites. Upon administration, questionnaires were checked for completeness and consistency by a member of the research team other than the interviewer. If missing information was detected, the interviewer was alerted and asked to retrieve it. This was possible most of the time since children were still at school after the interview.

### Assessment of school environment

Schools’ facilities, sanitary conditions, and deworming schedule were recorded using a standardized questionnaire which required interviewing school Principals as well as a visual inspection of the facilities by a researcher.

### Data management and statistical analysis

Data from questionnaires and Kato-Katz results were entered by a researcher into Microsoft Office Excel spreadsheet 2007 (Microsoft) and verified for accuracy (compared with data in questionnaires) by a different researcher. Data were checked for errors, missing values and extreme values or outliers. Statistical analyses were carried out using Stata 13 (College Station, TX: StataCorp LP) and IBM SPSS Statistics version 20.0 (Armonk, NY: IBM Corp.). Descriptive statistics (frequency, cumulative frequency, percentages, means and standard deviations) were used to characterize the study population. Point prevalences with 95% confidence intervals (95% CI) were calculated for overall STH infections and for each STH species. Similarly, point prevalences with 95% CI were calculated for monoparasitism and for mixed STH infections (*i.e*., infections with more than one species).

Risk factors were assessed through univariate and multivariable analyses. The defined outcomes of interest were: (i) *Ascaris lumbricoides* infection, (ii) *Trichuris trichiura* infection, (iii) hookworm infection, (iv) mixed infections, (v) moderate-to-heavy ascariasis, and (vi) moderate-to-heavy trichuriasis. As previously reported [19], *S. stercoralis* was not identified among the study population, precluding any analysis for this parasite.

For the regression models, moderate and heavy infections were merged into one category and compared to light infections since the public health importance of STH infections is generally associated with higher worm burdens [10]. Because the vast majority (94.1%) of hookworm infections were light; models for infection intensity were only constructed for *A. lumbricoides* and *T. trichiura.*

Based on the literature, a total of 15 variables (putative risk factors) were selected for testing their association with STH. Of these 15 variables, three were compound variables (*i.e.*, socio-economic status –SES–, STH awareness, and school hygienic condition); they were constructed as follows.

A household-based asset approach [24] was used to establish SES of the study participants. As recommended by the Health Nutrition and Population/Poverty thematic group of the World Bank, principal component analysis (PCA) was used to construct wealth quintiles, namely, (i) poorest, (ii) very poor, (iii) poor, (iv) less poor, and (v) least poor [25, 26]. Briefly, PCA was calculated from household assets (electricity, TV set, and refrigerator) and house characteristics (type of floor and type of sanitary facility). The first principal component explained 54% of the total variability. Locally weighted scatterplot smoothing (Lowess) was used to assess nonlinear relationship with the outcomes.

Determination of STH awareness was based on four criteria entailing a maximum score of five points. During the interview, children were asked if they were familiar with intestinal worms and if they thought they were at risk of getting infected with them. These questions had a dichotomous (yes/no) answer format and were assigned values of 1 = yes and 0 = no. Additionally, children’s knowledge of STH transmission and prevention was assessed using two open-ended questions. A value of 1.5 each was assigned to these questions since they probed a higher level of understanding. Children providing at least one correct answer to these questions received the respective full marks. Lowess plots of the data were used to assess nonlinear relationship with the outcomes.

School’s hygienic conditions were assessed through visual inspection of bathrooms and surrounding areas and ranked using a set of 10 hygiene indicators for a maximum score of 10 points. After Lowess plot analysis, the variable was dichotomized using a cut-off value of 6.0. Schools scoring below 6 were categorized as of lower hygienic level. The set of indicators recorded the presence or absence of desirable features such as access to water, sink, soap, functional latrine/toilet, toilet paper, cleanliness of toilet/latrine stall and surrounding areas, and odour control. Most features could be recorded with a yes/no answer and were assigned a value of 1 or 0, respectively. Two features (*i.e.*, type of sanitary facility and presence of a sink with water for handwashing) had three possible options, and in this case, values of 0, 0.5 and 1 were assigned to reflect absence, partial, or complete fulfillment, respectively.

Unadjusted odd ratios (OR) in the univariate analysis were calculated using logistic regression, whereas generalized estimating equations (GEE) multivariable models were used to estimate adjusted ORs. This approach accounts for the correlation among study participants attending the same school (school clustering effect). Based on likelihood-ratio (LR), a stepwise backward elimination of non-significant variables was used to find parsimonious models best predicting the outcomes, retaining those variables with *p*-values < 0.2.

## Results

### Study participation and population characteristics

**Figure 1:**
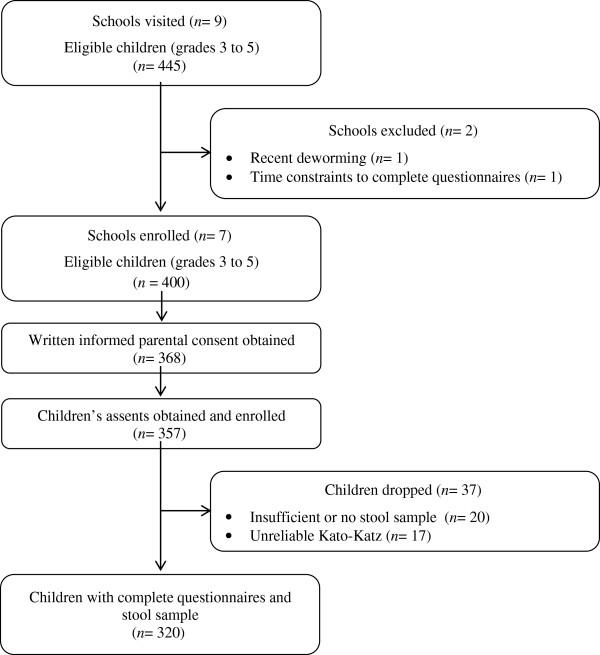
**Flow chart detailing the study recruitment process.** The investigation was conducted in 2011 and the study population comprised of children enrolled in seven schools located in rural communities of the Department of Olancho, Honduras.

The details of the study participation are shown in Figure 1. Seven of the nine visited schools were enrolled in the study. The two remaining schools, Santa Clara and Los Lirios, were excluded due to recent deworming treatment or time-constraints to complete questionnaires, respectively. The final study sample consisted of 320 children.

The characteristics and parasitological findings of the study sample are presented in Table 1. Participating children were aged 7–14 years (mean 9.76 ± 1.4) and 154 (48%) were girls. In terms of household conditions as reported by children, about 37% had partial or complete earthen floor, almost 50% had electricity, approximately 88% had either latrines or flush toilets, and 86% had piped water (usually a single faucet in the house’s backyard). Most children (90%) reported handwashing regularly and 28% acknowledged practicing habitual or occasional open defecation. More than half (58%) of respondents recalled expelling worms in the past but, in contrast, the vast majority (97%) did not consider themselves at risk of acquiring STH infections. Moreover, only 22% of children demonstrated good understanding of STH transmission and prevention.

**Table 1:** **Characteristics and parasitological findings of the study population enrolled in seven schools in rural Honduras (** ***n*** **= 320)**

Characteristics	***n***(%)
Age -*mean* (*SD*)	9.76 (1.4)
Girls	154 (48.1%)
**Household conditions**	
Earthen floor (complete or partial) (*n* = 317)^a^	118 (37.2%)
Electricity service	158 (49.8%)
Sanitary facility available	280 (87.5%)
Access to piped water	276 (86.3%)
**Practices and STH history**	
Habitual or occasional open defecation	91 (28.4%)
Reported regular handwashing	287 (89.7%)
Walked outdoor without shoes	163 (50.9%)
Performed chores outdoor	62 (19.4%)
Reported expelling worms in the past	186 (58.1%)
Recalled previous deworming (*n* = 310)^b^	275 (88.7%)
**Socio-economic status (** ***n*** **= 317)** ^**c**^	
Poorest	75 (23.7%)
Very poor	58 (18.3%)
Poor	61 (19.2%)
Less poor	60 (18.9%)
Least poor	63 (19.9%)
**Awareness and knowledge about STH**	
Lowest awareness	89 (27.8%)
Lower awareness	137 (42.8%)
Higher awareness	22 (6.9%)
Highest awareness	72 (22.5%)
**School hygienic conditions** ^**d**^	
Higher level	135 (42.2%)
**School deworming schedule**	
None or once a year	246 (76.9%)
Twice a year	74 (23.1%)
**Parasitic profile**	
Overall prevalence of STH infections	232 (72.5%)
*Ascaris lumbricoides*	97 (30.3%)
*Trichuris trichiura*	214 (66.9%)
Hookworms	51 (15.9%)
Mixed infections (*n* = 232)	103 (44.4%)
Moderate-to-heavy infections by *Ascaris lumbricoides* (*n* = 97)	58 (59.8%)
Moderate-to-heavy infections by *Trichuris trichiura* (*n* = 214)	57 (26.6%)
Moderate-to-heavy infections by hookworms (*n* = 51)	3 (5.9%)

STH: soil-transmitted helminth.

^a^Three children did not recall type of floor at home.

^b^Ten children did not recall receiving previous deworming treatment.

^c^Three missing cases. Socio-economic status score was calculated for 317 children.

^d^Schools obtaining a scores >6 out 10 were considered having higher level of hygiene.

### School environment

As per school Principals’ report, five of the seven participating schools had been providing deworming treatment using a single-oral dose of 400 mg albendazole, either twice a year (two schools) or once a year (three schools). The remaining two schools in which no deworming treatment had been administered in the last couple of years were smaller (with a combined enrolment of 86 children) and were farther away from Poncaya’s health centre than the other schools. The variation in deworming frequency was attributed by Principals to the availability of medication sent by the Ministry of Health to schools across the country.

In terms of hygienic conditions, only three schools obtained scores higher than the cut-off value (6.0) and were hence considered to have a higher level of hygiene compared to the rest. This meant that less than half (42%) of participating children were attending schools with higher levels of hygiene. At the time of the inspection, hand soap was available in one school; toilet paper was available in another. According to school Principals, the school rarely provided soap and toilet paper. Most schools had functional flush toilets, although they struggled with both waste disposal in surrounding areas and odour control.

### Parasitological findings

The parasitological profile of the study population has been reported elsewhere [19]. Briefly, no cases of *S. stercoralis* were found, and the combined prevalence of infection by any of the other helminth species was 72.5% (95% CI = 67.3 - 77.3). Prevalences for *A. lumbricoides*, *T. trichiura* and hookworms were 30.3% (95% CI = 25.3 – 35.7), 66.9% (95% CI = 61.4 – 72.0) and 15.9% (95% CI = 12.1 – 20.4), respectively (Table 1). According to egg counts, 40%, 73% and 94% of ascariasis, trichuriasis and hookworm infections were light-intensity. More than half (53.6%) of *A. lumbricoides* infections were of moderate intensity. Heavy-intensity infections were uncommon, representing 6.2%, 1.9%, and 3.9% of the cases of ascariasis, trichuriasis and hookworm infections, respectively. Almost half of the children (44.4%) had mixed infections, and 26% of them harboured all three STH species.

### Risk factors for STH infections

Results of the univariate analysis are shown in Table 2. Several putative risk factors were found significantly statistically associated with infections by individual STHs (notoriously in greater number for *A. lumbricoides*) as well as with mixed infections. Remarkably, associations between putative risk factors and infection intensity were more frequent for moderate-to-heavy trichuriasis than for ascariasis of similar intensity.

**Table 2:** **Univariate analysis of risk factors for STH infections in schoolchildren of rural Honduras (** ***n*** **= 320)**

									***A. lumbricoides***		***T. trichiura***	
Variables	***A. lumbricoides***		***T. trichiura***		Hookworms		Mixed infections		Mod-heavy (***n*** = 97)		Mod-heavy (***n*** = 214)	
	OR (95% CI)	***p***-value	OR (95% CI)	***p***-value	OR (95% CI)	***p***-value	OR (95% CI)	***p***-value	OR (95% CI)	***p***-value	OR (95% CI)	***p***-value
Age^a^	0.94 (0.78-1.12)	0.488	1.14 (0.97-1.35)	0.119	1.23 (0.99-1.52)	0.053	1.01 (0.85-1.21)	0.862	1.02 (0.78-1.34)	0.867	0.99 (0.79-1.24)	0.926
Sex (boys vs. girls)	1.40 (0.87-2.27)	0.168	1.06 (0.66-1.69)	0.815	2.07 (1.10-3.90)	0.023	1.64 (1.02-2.65)	0.041	1.55 (0.68-3.55)	0.295	1.02 (0.55-1.87)	0.959
SES^b^	0.73 (0.61-0.88)	0.001	0.77 (0.65-0.90)	0.002	0.83 (0.67-1.03)	0.093	0.75 (0.63-0.89)	0.001	0.96 (0.72-1.28)	0.790	0.80 (0.65-0.99)	0.044
Earthen floor at home (total-partial vs. cement-tile)	1.90 (1.17-3.11)	0.010	2.95 (1.73-5.05)	<0.001	1.48 (0.80-2.71)	0.207	1.96 (1.21-3.17)	0.007	1.60 (0.70-3.65)	0.267	1.85 (1.00-3.42)	0.050
Sanitary facility at home (none vs. latrine-toilet)	2.63 (1.34-5.17)	0.005	3.95 (1.50-10.42)	0.006	2.65 (1.24-5.65)	0.012	3.00 (1.53-5.90)	0.001	0.60 (0.22-1.63)	0.321	2.45 (1.15-5.21)	0.020
Piped water at home (no vs. yes)	2.15 (1.12-4.13)	0.021	2.48 (1.11-5.55)	0.027	0.81 (0.32-2.03)	0.654	1.94 (1.01-3.70)	0.045	1.01 (0.37-2.77)	0.983	0.50 (0.19-1.27)	0.145
Handwashing (occasionally vs. regularly)	2.08 (1.00-4.33)	0.050	3.04 (1.14-8.13)	0.027	1.19 (0.47-3.06)	0.711	1.88 (0.91-3.91)	0.089	0.73 (0.24-2.22)	0.582	1.36 (0.58-3.22)	0.482
Open defecation (occasional-habitual vs. no)	3.11 (1.86-5.20)	<0.001	3.03 (1.66-5.54)	<0.001	1.99 (1.07-3.70)	0.030	3.30 (1.98-5.50)	<0.001	0.95 (0.42-2.15)	0.898	2.04 (1.10-3.81)	0.024
Wearing shoes outdoors (no vs. yes)	1.67 (1.03-2.71)	0.038	1.25 (0.78-2.00)	0.344	1.21 (0.66-2.21)	0.538	1.64 (1.02-2.63)	0.042	1.80 (0.78-4.15)	0.165	2.71 (1.41-5.18)	0.003
Domestic chores (outdoor vs. indoor)	2.45 (1.39-4.35)	0.002	2.12 (1.09-4.12)	0.026	1.53 (0.76-3.10)	0.231	2.58 (1.46-4.56)	0.001	1.75 (0.69-4.43)	0.234	1.47 (0.73-2.94)	0.281
Recalled expelling worms (yes vs. no)	1.83 (1.11-3.02)	0.019	1.46 (0.91-2.34)	0.113	1.39 (0.74-2.59)	0.301	1.73 (1.06-2.82)	0.028	2.43 (1.01-5.85)	0.048	1.53 (0.80-2.91)	0.195
Recalled previous deworming (yes vs. no)^c^	0.71 (0.34-1.47)	0.354	1.57 (0.76-3.21)	0.219	0.76 (0.31-1.85)	0.549	0.78 (0.38-1.63)	0.513	1.79 (0.55-5.84)	0.337	0.88 (0.32-2.42)	0.807
STH awareness^d^	0.86 (0.67-1.11)	0.248	0.90 (0.72-1.11)	0.314	0.74 (0.55-1.00)	0.053	0.80 (0.63-1.01)	0.061	0.98 (0.69-1.40)	0.912	0.95 (0.71-1.28)	0.759
School hygiene (lower vs. higher level)	6.37 (3.46-11.73)	<0.001	4.75 (2.88-7.84)	<0.001	1.05 (0.57-1.93)	0.874	4.32 (2.49-7.48)	<0.001	2.60 (0.84-8.07)	0.098	1.27 (0.64-2.51)	0.491
School deworming (none-once vs. twice a year)	3.50 (1.71-7.17)	<0.001	0.81 (0.46-1.43)	0.480	3.17 (1.21-8.32)	0.019	3.89 (1.90-7.96)	<0.001	0.99 (0.26-3.79)	0.989	5.83 (1.99-17.08)	0.001

OR: odds ratio; CI: confidence interval; STH: soil-transmitted helminth; SES: socio-economic status; Mod-Heavy: moderate-to-heavy infections.

^a^Age as continuous variable in years.

^b^SES quintiles calculated as described in methodology section. Three missing data (*n* = 317).

^c^Ten children did not recall receiving previous deworming treatment.

^d^STH awareness score calculated as described in methodology section. Range = 0 – 5.

Estimated ORs from multivariable logistic regression models of species-specific prevalences, mixed infections prevalence, and infection intensity are presented in Table 3.

**Table 3:** **Multivariable logistic models of STH infections using Generalized Estimating Equations to account for within-school clustering**

									***A. lumbricoides***		***T. trichiura***	
	***A. lumbricoides***		***T. trichiura***		Hookworms		Mixed infections		Mod-heavy		Mod-heavy	
	(***n*** = 307)		(***n*** = 317)		(***n*** = 320)		(***n*** = 310)		(***n*** = 96)		(***n*** = 211)	
Variables (Risk category)	OR (95% CI)	***p***-value ^a^	OR (95% CI)	***p***-value ^a^	OR (95% CI)	***p***-value ^a^	OR (95% CI)	***p***-value ^a^	OR (95% CI)	***p***-value ^a^	OR (95% CI)	***p***-value ^a^
Age^b^	0.84 (0.67-1.05)	0.120	1.14 (0.92-1.43)	0.226	1.21 (0.96-1.53)	0.107	--		--		0.85 (0.65-1.09)	0.206
Sex (Boys)	1.64 (0.90-3.00)	0.106	--		2.33 (1.23-4.42)	0.010	2.09 (1.17-3.75)	0.013	--		--	
SES (Lower)^c^	0.80 (0.65-0.99)	0.039	0.77 (0.63-0.94)	0.010	--		--		--		--	
Earthen floor at home (Total or partial)	--		--		--		--		1.87 (0.77-4.55)	0.166	2.12 (1.05-4.29)	0.036
Home sanitary facility (None)	--		--		3.23 (1.37-7.57)	0.007	--		--		2.30 (0.94-5.64)	0.068
Handwashing (Occasionally)	2.56 (0.96-6.79)	0.060	2.70 (0.93-7.81)	0.067	--		--		--		--	
Open Defecation (Habitual or occasional)	--		--		--		1.78 (0.97-3.27)	0.064	--		--	
Wearing shoes outdoors (No)	--		--		--		--		--		3.44 (1.73-6.87)	<0.001
Recalled expelling worms (Yes)	--		--		--		--		3.00 (1.19-7.55)	0.020	--	
Recalled deworming (Yes)^d^	0.37 (0.14-0.96)	0.042	--		--		0.55 (0.23-1.31)	0.178	--		--	
STH awareness (Lower)^e^	--		--		0.72 (0.52-0.99)	0.040	0.75 (0.57-0.98)	0.036	--		--	
School deworming (None or only once a year)	10.40 (4.39-24.65)	<0.001	2.08 (0.97-4.45)	0.067	2.92 (1.09-7.85)	0.034	10.57 (4.53-24.66)	<0.001	--		8.12 (2.33-28.20)	0.001
School hygiene (Lower level)	14.85 (7.29-30.24)	<0.001	7.32 (3.71-14.45)	<0.001	--		9.02 (4.66-17.46)	<0.001	3.32 (1.05-10.52)	0.041	--	

OR: odds ratio; CI: confidence interval; STH: soil-transmitted helminth; SES: socio-economic status.

^a^ *p*-value from likelihood ratio test.

^b^Age as continuous variable in years.

^c^SES quintiles calculated as described in Methods. Three missing data (*n* = 317).

^d^Ten children did not recall receiving previous deworming treatment.

^e^STH Awareness score calculated as described in Methods. Range = 0 – 5.

(--) Variable not included in the final model.

The age of children was not found significantly associated with STH infection. However, as age increased by one year, the odds for ascariasis were reduced by about 20%. Conversely, we observed that a one-year increase in children’s age increased the odds for *T. trichiura* and hookworm infections by 15% and 20%, respectively.

Sex was only found significantly associated with hookworm infection and with having mixed infections. Boys had twice the odds of both being infected by this parasite (OR = 2.33, 95% CI = 1.23 – 4.42, *p* = 0.010) and harbour multiple STH infections (OR = 2.09, 95% CI = 1.17 – 3.75, *p* = 0.013).

Higher SES had a protective effect against *A. lumbricoides* (OR = 0.80, 95% CI = 0.65 - 0.99, *p* = 0.039) and *T. trichiura* (OR = 0.77, 95% CI = 0.63 - 0.94, *p* = 0.010). As their household SES improved by one quintile (*e.g.,* from very poor to poor), children’s odds of being infected with these two parasites decreased by 20%.

Children living in households with earthen floors were at increased odds of harbouring moderate-to-heavy trichuriasis (OR = 2.12, 95% CI = 1.05 - 4.29, *p* = 0.036).

Children living in households lacking toilets or latrines had increased odds of hookworm infection (OR = 3.23, 95% CI = 1.37 - 7.57, *p* = 0.007). They were also at increased risk of harbouring moderate-to-heavy trichuriasis although, in this case, statistical significance was only marginal (OR = 2.30, 95% CI = 0.94 - 5.64, *p* = 0.068).

When compared to children reporting regular handwashing, those describing occasional practice had about three times higher odds of harbouring infections by *A. lumbricoides* and *T. trichiura,* although statistical significance was only marginal (*p* = 0.060 and *p* = 0.067, respectively).

Likewise, children practicing habitual or occasional open defecation had almost twice the odds of being parasitized with more than one STH species. This association too was only marginally significant (OR = 1.78, 95% CI = 0.97 - 3.27, *p* = 0.064).

Not wearing shoes outdoors was associated with moderate-to-heavy trichuriasis (OR = 3.44, 95% CI = 1.73 - 6.87, *p* < 0.001).

Children who recalled expelling worms in the past had three times the odds of having moderate-to-heavy ascariasis (OR = 3.00, 95% CI = 1.19 - 7.55, *p* = 0.020), and those who recalled receiving deworming treatment had reduced odds of ascariasis (OR = 0.37, 95% CI = 0.14 - 0.96, *p* = 0.042) and mixed infections. For the latter, though, such association did not reach statistical significance (*p* = 0.178).

Increased awareness of STH transmission played a significantly protective role against harbouring mixed infections (OR = 0.75, 95% CI = 0.57 - 0.98, *p* = 0.036) and hookworms (OR = 0.72, 95% CI = 0.52 - 0.99, *p* = 0.040), but not against the other two species.

The school environment was found to play an important role in STH infections. School deworming frequency was significantly inversely associated with ascariasis, hookworm infection, and multiple parasite infections. Children attending schools with absent or once-a-year deworming schedules had increased odds of being infected by *A. lumbricoides* (OR = 10.40, 95% CI = 4.39 - 24.65, *p* <0.001), of harbouring hookworm infections (OR = 2.92, 95% CI = 1.09 - 7.85, *p* = 0.034), and of harbouring mixed infections (OR = 10.57, 95% CI = 4.53 - 24.66, *p* <0.001). In contrast, the association between deworming schedule and *T. trichiura* infections was only marginally significant (OR = 2.08, 95% CI = 0.97 - 4.45, *p* = 0.067).

Children in schools with absent or once-a-year deworming schedules had significantly increased odds of having moderate-to-heavy trichuriasis (OR = 8.12, 95% CI = 2.33 - 28.20, *p* < 0.001).

School hygienic conditions were significantly inversely associated with the prevalence of *A. lumbricoides* and *T. trichiura* infections as well as with the prevalence of mixed infections. Children attending schools with a lower level of hygiene had increased odds for ascariasis (OR = 14.85, 95% CI = 7.29 - 30.24, *p* <0.001), trichuriasis (OR = 7.32, 95% CI = 3.71 - 14.45, *p* <0.001) and for harboring mixed infections (OR = 9.02, 95% CI = 4.66 - 17.46, *p* <0.001). Similarly, low hygienic conditions in the schools were significantly associated with higher intensity infections with *A. lumbricoides* (OR = 3.32, 95% CI = 1.05 - 10.52, *p* = 0.041).

## Discussion

Despite the ubiquitous nature of childhood soil-transmitted helminthiases in Honduras [20, 27], research on this topic in the country is limited. Here we present an in-depth investigation of the risk factors playing a role in the prevalence of STH infections in Honduran children living in endemic rural communities. In the univariate analysis, a series of factors were found significantly statistically associated with STH infection, but once controlling for possible confounders and school cluster effect, the strength of association for some factors decreased. Thus, the following discussion pertains only to the risk factors included in the multivariable logistic models.

### Children’s age

Although it is widely known that increased age is a protective factor for both *A. lumbricoides* and *T. trichiura* infections [28–32] and a risk factor for hookworm infections [33–38], this was not observed in our study population. Such a finding may be due to the narrow age range of the participating children (7–14 years). In fact, our study identified a consistent trend in the protective effect of age over *A. lumbricoides* infection, and conversely, as a risk factor for hookworm infection. Unexpectedly, and as found by a recent study in Laos [39], we observed that as the age of children increased, the odds of having trichuriasis also increased. We could not find a plausible explanation in the literature for this finding and can only suggest assessing its validity through larger studies and/or longitudinal studies including wider population age groups. It would be also worth evaluating whether deworming treatment has any differential effect on the age distribution of reinfections.

### Sex of the children

We found that the odds of having mixed infections were significantly higher in boys than in girls. Also, being a boy was significantly associated with increased prevalence of hookworm infection. However, boys in our study did not report outdoor activities or barefoot walking more frequently. Studies on hookworm infections worldwide have consistently identified higher infection prevalence in males [34, 37, 38, 40–43] but so far, it is unclear whether this finding is due to differential exposure [44], physiological factors [45, 46] or a combination of both [33, 47]. Future STH investigations in Honduras need to pay closer attention to sex and gender differentials and evaluate the impact of social roles and behaviours in terms of STH infection and reinfection.

### Family socio-economic status (SES)

Significantly lower odds of having ascariasis and trichuriasis (but not hookworms) were found in children belonging to families in the upper SES quintiles. It has been proposed that *A. lumbricoides* and *T. trichiura* are mainly transmitted within the domestic domain whereas hookworms are transmitted in the public domain [48, 49]. Hence, increases in family SES may have a direct effect in preventing both ascariasis and trichuriasis but not infection by hookworm. In general, our findings coincide with other studies conducted worldwide [26, 39, 41, 50–53] and show that even in communities where poverty seems to affect everyone equally, slight variations in wealth within households may be able to exert a protective effect against STH infections. Interestingly, as discussed above, the specific absence of sanitary facilities in the homes was not statistically associated with *A. lumbricoides* or *T. trichiura*. Altogether our data may suggest that beyond material possessions, a higher SES may also represent increased level of education among family members, increased hygiene practices, or perhaps both. Data on parents education or family composition was not collected by our survey and this is a gap that needs to be addressed in future investigations.

### Sanitary facilities in the home

Only 12.5% of research participants lived in households without an adequate option for feces disposal. Our findings are congruent with data from the Honduran government stating that 15.5% of rural homes are in a similar situation (http://www.ine.gob.hn/index.php/datos-y-estadisticas/estadisticas-sociales-y-demograficas/vivienda/69-acceso-a-sevicios-basicos, accessed July 17, 2014). This lack of sanitary facilities increased children’s risk of acquiring hookworm infections by an order of three compared to children with access to at least a latrine at home. Comparable studies in Guinea-Bissau [38], Burkina-Faso, Ghana and Mali [37] have made similar observations. An explanation proposed for these findings is that in the absence of toilets or latrines, children return regularly to the defecation site, where viable, infectious hookworm larvae remain and the children are therefore continually exposed to reinfection [54]. Linking this finding with the above-mentioned increased odds of hookworm infection in boys, it would be worthwhile investigating whether boys have more consistent behaviour patterns than girls. This was not assessed by the present study. In terms of *A. lumbricoides* or *T. trichiura*, unlike other studies [55–58], our data did not support a statistical association between these two species and the absence of sanitary facilities in the homes.

### Handwashing

Our data show a marginal statistical significant difference between children practicing only occasional handwashing versus those reporting handwashing regularly: they had almost a three times higher odds of being infected by *A. lumbricoides* and *T. trichiura.* A large body of literature supports access to clean water and handwashing as cost-effective and important hygiene measures in preventing the spread of infectious diseases in general [59–61] and intestinal helminths in particular [34, 39, 41, 62, 63]. A major review carried out in 2009 by Fung and Cairncross shows that studies (most relying on self-reporting) have found disparate results when assessing the association of handwashing with the prevalence and infection intensity of *A. lumbricoides* [63]. Inconsistencies between self-reported handwashing versus actual observation have been documented by other authors [64]. Altogether these findings underscore the need for studies to use reliable, perhaps standardized indicators when characterizing hygiene-related habits and attitudes.

### Open defecation (OD)

About 28% of children acknowledged practicing either habitual or occasional OD. This figure seems excessive since only 12.5% of children reported not having at least a latrine at home. However, it is important to clarify that information in regard to actual usage or functionality of sanitary facilities at home was not collected by our survey. Therefore, it is entirely possible that not all children were able or willing to use the latrine or toilet in the home or school. Conversely, in view of the personal nature of the question, it is also possible that not all children practicing OD acknowledged such practice [65]. These inconsistencies in the data and gaps might explain why our study could not identify OD as a risk factor for STH infections. However, other similar studies were not able to demonstrate this particular association either [14, 34, 62].

### Self-reported worm expulsion and deworming history

It was interesting to find a statistically significant association between a past history of expelling intestinal worms (likely *A. lumbricoides* adults, due to their size) and the odds of having a moderate-to-heavy infection by this parasite. It is possible that heavily infected children are regularly passing worms and are thus more likely to notice and remember; they may also have more concerns and might be more likely to report it. This finding suggests that self-reporting might be a good indicator for finding heavily infected children who merit individual attention and health care. Our study also found that a history of previous deworming as reported by children was significantly associated with a 64% reduction in the odds of having ascariasis but not the other two infections. This result is important for two reasons. Firstly, it hints to the greater effectiveness of preventive chemotherapy (PC) with single-dose albendazole against *A. lumbricoides* than for the other two STH species [66, 67]. Secondly, it reveals that Honduran rural children are very familiar with deworming treatment and are able to reliably recall if deworming tablets have been provided to them at school. The latter could be used as a rapid monitoring tool for treatment uptake, which, combined with strategic prevalence studies, could provide a more complete idea of the country’s situation.

### STH awareness

Increasing the level of awareness about STH –how parasites are acquired, their importance, and what it means for the host to harbour them– has proved effective in preventing STH infections [68]. Accordingly, health education is suggested as a fundamental component of comprehensive STH control programs [10, 69–72]. In the studied children, level of awareness showed a significant protective effect against hookworm infections probably because these tend to occur at greater frequency in older children, who in turn may have greater awareness than younger children. This explanation, however, remains speculative as our data show find a strong association between age and hookworm infection.

### Hygiene conditions in the schools

A major finding from this study was that the hygiene conditions in the schools played an important role in the transmission of both *A. lumbricoides* and *T. trichiura*, and also in the prevalence of mixed infections. Further, independently of other factors, children enrolled in the schools with lower hygiene levels were more likely to harbour higher intensity infections by *A. lumbricoides*. There is no doubt that improvements to water, sanitation, and hygiene (WASH) contribute to a significant reduction in STH transmission [73, 74] but empirical evidence of this positive association is not always demonstrated. A recent meta-analysis was just able to show that WASH access and practices at the community level were associated with reduced odds of STH infection [75]. At the school level, some cross-sectional studies have shown that poor school sanitation increased the risk for parasitic infections [76]. More recently, a cluster-randomized trial undertaken in Kenya, demonstrated that school hygiene and sanitation reduced the re-infection prevalence of *A. lumbricoides* [77]. Our study is the first in Honduras to investigate the role of school hygiene on STH transmission and our results can perhaps open the discussion for greater involvement by school teachers. School teachers have already proven to be valuable aides in STH control programs [78]. As direct lines of communication with pupils, school authorities, and parents, teachers could become instrumental advocates for cleaner, more hygienic schools thus further preventing opportunities for STH transmission [79]. Given that they spend between 5 to 6 hours a day in school, such improvements may result in significant health gains for children.

### School deworming schedule

A second major finding of this study was that frequency of deworming in the schools was strongly associated with lower STH prevalence. It was also associated with lower intensity of *T. trichiura* infections. This is good news for the Honduran government as it provides evidence that current efforts are helping to achieve control targets (http://presencia.unah.edu.hn/vinculacion/articulo/desparasitaran-a-dos-millones-de-escolares- accessed July 17, 2014). On the other hand, our study revealed that operational challenges remain, as, despite geographical proximity, not all schools had administered PC in the last couple of years. Finding that children who had not received PC had similar odds of being infected by *A. lumbricoides* and hookworms, as well as of being infected by multiple STH species than children receiving PC once a year, lends support to the decision by the Honduran Ministry of Health of increasing the frequency of PC administration, as suggested by international health governing bodies [10, 80, 81]. While it is recognized that PC alone will not eliminate STH infections [82, 83], administered at the appropriate frequency, it can decrease the burden and morbidity of these parasitoses [84]. Finally, the frequency of deworming in the schools showed only a marginally significant effect in *T. trichiura* prevalence, which is likely due to the low efficacy of single-dose albendazole/mebendazole against this parasite [66, 67, 85]. This might explain why this and other Honduran studies show that *T. trichiura* is becoming the predominant STH species in the country [20].

Examining the school environment by assessing both the physical sanitary conditions as well as the schools deworming practices revealed crucial information and our results lend support to WHO’s Health promoting schools (HPS) initiative [86]. In terms of STH infections, greater consideration should be given to schools as either potential enablers of transmission or as crucial players in their control.

It is worth emphasizing that we did not implement techniques sensitive enough for reliable *S. stercoralis* detection. Although the personnel performing the Kato-Katz examination were experienced and would have been able to identify helminth larvae in fecal smears, had they been present, the possibility of underdiagnosis due to the method’s low sensitivity cannot be ruled out. Regrettably, there is no reliable prevalence data for *S. stercoralis* in Honduras. A national survey carried out by the Ministry of Health in 2011 also utilized the Kato-Katz method. A sample of 2,554 schoolchildren enrolled in 48 schools across the nation were examined and a prevalence of 0.4% for *S. stercoralis* was found (while prevalences for *A. lumbricoides*, *T. trichiura* and hookworms were 22.3%, 34% and 0.9%, respectively) [87]. In Honduras, as in many countries, *S. stercoralis* is rarely recognized as a public health problem [88], and future studies should strive to integrate its diagnosis.

There are some limitations to the present study. Since the study population was obtained through the parent study, which aimed to assess gender-specific risk behaviours in relation to STH prevalence (manuscript in preparation), our sample size was not calculated with our primary outcomes in mind and this may have somewhat limited the power of the study. Reporting biases might have been introduced while collecting information from participating children (*e.g.,* household SES data; self-reporting of hygiene practices such as handwashing, OD, usage of shoes). Also, information on parents’ level of education was not obtained and such data might have been important when constructing the SES variable. There are also potential limitations in terms of the diagnostic methodology utilized in this study. The time elapsed between sample production by research participants and smear preparation was approximately six hours, and as noted by other authors, some hookworm eggs and *S. stercoralis* larvae may have deteriorated, thus limiting diagnostic sensitivity and resulting in under-reporting [89]. However, the prevalence of hookworm infections obtained in our study was 15.9%, a much higher figure than the 0.9% national prevalence reported in the last survey conducted by the Ministry of Health in 2011. Further, more relevant to our study, the national survey included examining 230 school children 9–11 years of age living in rural communities of Olancho, the same Department where the present study was conducted, and found a prevalence of 0% [90]. Our success in detecting hookworm eggs may be due to having stored samples under cool conditions until smear preparation. Other authors have found that hookworm eggs were best preserved by keeping samples on ice or covered with a wet tissue [91].

Finally, given that this was a cross-sectional study in a defined geographical area, our findings might not be generalizable to other ecological zones in the country.

Despite these limitations, our study also possesses important strengths, for example, a high participation rate, the representativeness of our study participants of the rural population of Honduras, [18], and the use of the Kato-Katz method, recommended by the WHO for STH epidemiological surveys. Even though the analysis of a single-stool sample by a single diagnostic method might have underestimated prevalence and/or intensity of infection [23], in light of the high prevalence obtained, this underestimation might be minimal. Other authors concur that the Kato-Katz method can yield accurate results with one day’s stool collection for both *A. lumbricoides* and *T. trichiura*, although less so for hookworm eggs [92, 93]. An important strength of this study stems from the fact that we utilized robust statistical methods and modelled risk factors considering plausible variables for STH transmission taking into account within-school clustering.

## Conclusions

The findings presented here demonstrate that Honduras’s STH control efforts are going in the right direction. They also emphasize the need for continued PC administration, uptake and monitoring across the territory, paying particular attention to remote rural communities. In addition, our data suggests that remaining vigilant of STH prevalence trends, especially for *T. trichiura,* would be useful for assessing PC efficacy and assuring timely detection of drug resistance.

In light of the risk factors we found associated with STH infections, our findings also support the need for an integrated approach to STH control in Honduras [51, 80, 94, 95]; one that leads to resource optimization among different sectors to improve health outcomes [84]. An integrated approach is, in fact the country’s goal, as expressed in the “National strategic plan for the prevention, attention, control, and elimination of neglected infectious diseases in Honduras, 2012-2017” (PEEDH) [87]. However, the ability to address these factors in a comprehensive and integrated manner may be compromised by the reality of Honduras as a developing country [84]. Therefore, more feasible interventions need to be considered at the moment. We propose that along with the uninterrupted bi-annual deworming treatment of both preschool-age and school-age children, dedicated actions are taken to improve the hygienic conditions of the schools where Honduran children learn and play.

## Abbreviations

CI: Confidence interval
EPG: Eggs per gramme (of stool)
GEE: Generalized estimating equations
OR: Odds ratio
PAHO: Pan American Health Organization
PC: Preventive chemotherapy
SES: Socio-economic status
STH: Soil-transmitted helminth
UNA: Universidad Nacional de Agricultura (National University of Agriculture)
UNAH: Universidad Nacional Autónoma de Honduras (National Autonomous University of Honduras)
WHO: World Health Organization.
